# Measurement of back-scattered radiation from micro multileaf collimator into the beam monitor chamber from a dual energy linear accelerator

**DOI:** 10.4103/0971-6203.33243

**Published:** 2007

**Authors:** K. R. Muralidhar, P. Narayana Murthy, N. V. N. M. Sresty, Pramod Kumar Dixit, Rajneesh Kumar, A. K. Raju

**Affiliations:** Department of Physics, Nagarjuna University, Guntur, India; Department of Physics, Indo-American Cancer Institute and Research Center, Banjara Hills, Hyderabad, Andhra Pradesh, India

**Keywords:** Backscattered radiation, beam monitor chamber, micro multileaf collimator

## Abstract

Measurements designed to find the collimator backscatter into the beam monitor chamber from Micro Multileaf collimator of 6 MV photon beams of the Siemens Primus linear accelerator were made with the help of dose rate feedback control. The photons and electrons backscattered from the upper and lower secondary collimator jaws give rise to a significant increase in the ion charge measured by monitor chamber. This increase varies between the different accelerators. The output measurements were carried out in air at the isocenter. The effect of collimator backscatter was investigated by measuring the pulse width, number of beam pulses per monitor unit, monitor unit rate and dose for different mMLC openings. These measurements were made with and without dose rate feedback control, i.e., with constant electron beam current in the accelerator. Monitor unit rate (MU/min) was almost constant for all field sizes. The maximum variation between the open and the closed feedback control circuits was 2.5%. There was no difference in pulse width and negligible difference in pulse frequency. Maximum value of backscattered radiation from the micro Multileaf collimator into the beam monitor chamber was found to be 0.5%.

Bremsstrahlung x-rays are produced when the electrons are incident on a target of high Z-material such as tungsten. The treatment head consists of an x-ray target, scattering foil, flattening filter, ion chamber, fixed and movable collimators and light localization system. The treatment beam is first collimated by a fixed primary collimator located immediately beyond the x-ray target. In case of x-rays, collimated beam then passes through the flattening filter. The flattened x-ray beam is then incident on the dose monitor chamber.[1]

In an accelerator, the photon beam delivery is controlled by the signal from the beam monitor chambers through a feedback mechanism. The monitor chamber collects the ion charge produced within its air volume. When the total charge collected by the chamber corresponds to the required monitor signal settings, the interlock mechanism terminates the treatment.[2] The charge measured by the monitor chamber is mainly due to the forward scatter from the central section of the flattening filter and forward scatter from the collimators.[3]

Several studies[4–12] have indicated that there is an additional charge measured by the beam monitor chamber due to photons and electrons backscattered from the upper and lower secondary collimator jaws. The presence of backscattered photons and electrons from high atomic number materials has been experimentally investigated for Co-60 and 8 MV photon beams. Measurements showed that monitor chamber readings were increased by 20% for an 8 MV photon beam when a lead scatter was placed 10 cm below the chamber for a 15 × 15 cm^2^ field.[9]

Studies[6,10] have indicated that backscatter effect from a collimator is negligible for the varian clinac-1800 due to the absorption of the back-scattered photons by the finite thickness of the aluminum exit window. Measuring the magnitude of such contributions for a particular accelerator under specific operating conditions is therefore important to understand and model accelerator head factor.

In the present work, we have investigated the extent of backscatter into the beam monitor chamber from a fixed field of secondary collimator opening with different fields of attached mMLC for 6 MV photon beams from linear accelerator.

## Materials and Methods

The Siemens Primus linear accelerator has two photon energies (6 MV and 15 MV) and five different electron energies (6, 9, 12, 15, 18 and 21 MeV). The upper and lower jaws are located at about 22.47 cm and 30.27 cm away from the beam monitor chambers respectively. The Acculeaf (mMLC) is an external attachment to linear accelerator, which is at a distance of 56 cm from the target.

Siemens Primus linear accelerator with detachable micro Multileaf collimator (Acculeaf) is used to treat stereotactic radiotherapy, stereotactic radiosurgery and three-dimensional conformal radiotherapy cases. The linear accelerator is equipped with a xenon beam monitoring chamber which has a ceramic metal coating at the exit window.

**Figure 1:**
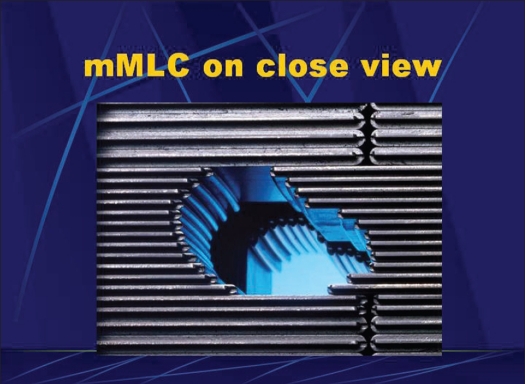
Micro multileaf collimator (Acculeaf) From Direx systems, UK

The effect of backscatter from mMLC was estimated by measuring the relative charge, beam pulse width, pulse frequency and monitor unit rate, keeping constant the field size of collimator at 10 × 10 cm^2^ and varying the field size of mMLC. Micro MLC (Acculeaf) was procured from Direx Systems Ltd. (U.K.), with four banks having X_1_, X_2_, Y_1_ and Y_2_. Each bank consisted of 24 leaves. Fourteen inner leaves had a leaf width of 3.1 mm and 10 outer leaves had a leaf width of 5.3 mm at isocenter, as shown in Figure 1.

**Figure 2:**
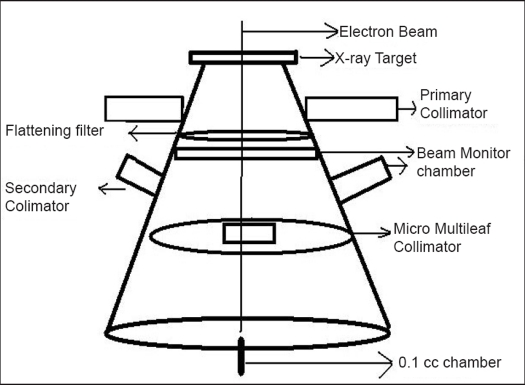
Measurement setup to estimate the backscatter effect of mMLC on output factors. The output factors were taken by keeping secondary collimator field size constant (10×10 cm^2^) and with variable field sizes of mMLC

The output measurements were done using Scanditronix Wellhofer's compact chamber CC01 and Dose-1 Reference Grade Therapy dosimeter for 6 MV photon energy. The chamber was positioned at the isocenter in air [Figure 2]. The field sizes of mMLC ranged from 1 × 1 cm^2^ to 10 × 10 cm^2^. The fixed field size of secondary collimator was 10 × 10 cm^2^.

The principle is that in the absence of dose rate feedback control, the electron beam current at the target and subsequently the photon output are constant. Monitor unit rate would be different for different positions of the collimators if the amount of collimator backscatter radiation were to change with field size. The amount of backscatter should increase as field size decreases in the presence of micro Multileaf collimator backscatter.[8]

Siemens Linear accelerators have dose rate feedback control circuits. They are activated in ‘closed loop’ and inactivated by ‘open loop’ operation. In the open loop mode, the electron beam current is independent of any variation in the dose rate measured by the dose monitor chamber. However, when the Siemens machines are operated in the closed loop mode, the ionization current from the dose monitor chamber is used to regulate electron beam current and photon output. An increase in the amount of collimator backscatter radiation would then cause a decrease in electron beam current and photon output.

**Figure 3:**
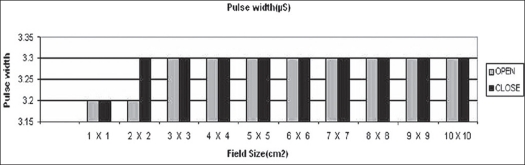
Beam pulse width in micro seconds (μS) for 1×1 cm^2^ to 10×10 cm^2^ field sizes in open and closed loops of dose rate feedback control

**Figure 4:**
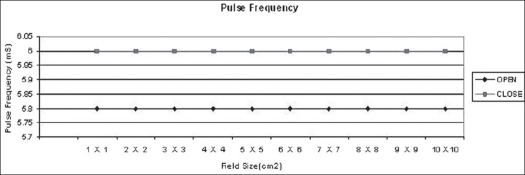
Pulse frequency in milli seconds (mS) for 1×1 cm^2^ to 10×10 cm^2^ field sizes in open and closed loops of dose rate feedback control

**Figure 5:**
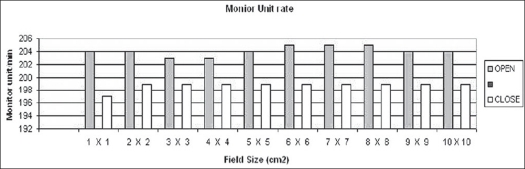
Monitor unit rate (MU/min) for 1×1 cm^2^ to 10×10 cm^2^ field sizes in open and closed loops of dose rate feedback control

**Figure 6:**
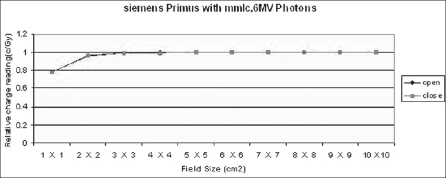
Results (Relative charge reading) with 6 MV X-ray beam from Siemens Primus Linear accelerator in open and closed loops of dose rate feedback control

In the open loop mode, monitor unit rates (MU/min), beam pulse rates and beam pulse widths and output factors were measured for 6 MV photon energies for field sizes ranging from 1 × 1 cm^2^ to 10 × 10 cm^2^. Charge readings were normalized at field size of 10 × 10 cm^2^. Beam pulse rates and beam pulse widths were measured by oscilloscope. The same measurements were taken in closed loop mode for all field sizes [Figures 3–6].

## Discussion

The technique described here is one of the methods to detect collimator backscatter radiation. This technique is not applicable to all therapy machines. The Siemens machine has feedback control facility. Reduction of 2.5% and 4% in dose delivered is observed due to backscatter radiation (BSR) measured from clinac 2100c.[13] With the use of telescopic technique in Clinac-1800 linear accelerator, the backscatter radiation by 6 and 15 MV photons is contribute to the field size dependent output by less than 1% and 2% respectively for square fields.[6] In the present study, we find 0.5% contribution due to BSR from mMLC.

## Results and Conclusions

It can be concluded that the maximum reduction of 0.5% in dose delivery is observed for 6 MV photons with constant secondary collimator field size and with various field sizes of mMLC. This is due to back-scattered radiation originating mainly from the mMLC reaching the beam monitor chamber. Monitor unit rate was almost constant for all field sizes. The maximum variation in monitor unit rate between open and closed feedback control circuits is 2.5%. There is no difference in pulse width and negligible difference in pulse frequency. Collimator backscatter radiation can be taken into account in dose calculation by a simple multiplicative factor. Hence we should take beam measurements very carefully while using detachable mMLC because of the contribution of its radiation backscatter effect into beam monitor chambers delivered.
